# Magnetic Levitation of Personalized Nanoparticle–Protein Corona as an Effective Tool for Cancer Detection

**DOI:** 10.3390/nano12091397

**Published:** 2022-04-19

**Authors:** Erica Quagliarini, Luca Digiacomo, Damiano Caputo, Alessandro Coppola, Heinz Amenitsch, Giulio Caracciolo, Daniela Pozzi

**Affiliations:** 1 NanoDelivery Lab, Department of Molecular Medicine, Sapienza University of Rome, Viale Regina Elena 291, 00161 Rome, Italy; erica.quagliarini@uniroma1.it (E.Q.); luca.digiacomo@uniroma1.it (L.D.); giulio.caracciolo@uniroma1.it (G.C.); 2 Department of Surgery, University Campus Bio-Medico di Roma, Via Alvaro del Portillo 200, 00128 Rome, Italy; d.caputo@policlinicocampus.it; 3 General Surgery, Fondazione Policlinico Universitario Campus Bio-Medico, Via Alvaro del Portillo 200, 00128 Rome, Italy; a.coppola@policlinicocampus.it; 4 Institute of Inorganic Chemistry, Graz University of Technology, Stremayrgasse 9/IV, 8010 Graz, Austria; amenitsch@tugraz.at

**Keywords:** nanoparticles, bio-nano interactions, protein corona, magnetic levitation, cancer detection, cancer nanotechnology

## Abstract

Unprecedented opportunities for early stage cancer detection have recently emerged from the characterization of the personalized protein corona (PC), i.e., the protein cloud that surrounds nanoparticles (NPs) upon exposure to a patients’ bodily fluids. Most of these methods require “direct characterization” of the PC., i.e., they necessitate protein isolation, identification, and quantification. Each of these steps can introduce bias and affect reproducibility and inter-laboratory consistency of experimental data. To fulfill this gap, here we develop a nanoparticle-enabled blood (NEB) test based on the indirect characterization of the personalized PC by magnetic levitation (MagLev). The MagLev NEB test works by analyzing the levitation profiles of PC-coated graphene oxide (GO) NPs that migrate along a magnetic field gradient in a paramagnetic medium. For the test validation, we employed human plasma samples from 15 healthy individuals and 30 oncological patients affected by four cancer types, namely breast cancer, prostate cancer, colorectal cancer, and pancreatic ductal adenocarcinoma (PDAC). Over the last 15 years prostate cancer, colorectal cancer, and PDAC have continuously been the second, third, and fourth leading sites of cancer-related deaths in men, while breast cancer, colorectal cancer, and PDAC are the second, third and fourth leading sites for women. This proof-of-concept investigation shows that the sensitivity and specificity of the MagLev NEB test depend on the cancer type, with the global classification accuracy ranging from 70% for prostate cancer to an impressive 93.3% for PDAC. We also discuss how this tool could benefit from several tunable parameters (e.g., the intensity of magnetic field gradient, NP type, exposure conditions, etc.) that can be modulated to optimize the detection of different cancer types with high sensitivity and specificity.

## 1. Introduction

Achieving early diagnosis is an essential element in the fight against cancer. Thus, the development of rapid and robust diagnostic tools is crucial in national healthcare systems, since it enables healthcare providers to minimize the mortality of the disease and improve patients’ quality of life [1]. However, despite the great attention from the scientific community, reaching an effective early diagnosis is still a big challenge. In fact, to date, there are a very limited number of non-invasive screening tests for early stage cancer detection for most cancer types. These include prostate-specific (PSA) tests for prostate cancer [2], colonoscopies for colorectal cancer [3], mammograms for breast cancer [4], and low-dose computerized tomography (LDCT) scans for lung cancer [5]. However, they lack specificity and sensitivity, require complex medical procedures, or have high costs for large-scale production. On the other hand, ongoing research on finding potential cancer biomarkers is showing promising results [6]. For example, protein biomarkers in the blood serve as molecular signatures of several diseases, including cancer, cardiovascular disorders, and other pathological conditions [7]. However, a biomarker may be present at a very low concentration (e.g., sub-ng/mL) in the blood, which requires large volumes of samples, a long processing time for classical biomarker analysis techniques (e.g., enzyme-linked immunosorbent assay (ELISA)) and high costs, making their applicability possible only in contexts with high resources [8]. These requirements do not meet the ASSURED (Affordable, Sensitive, Specific, User-friendly, Rapid and robust, Equipment-free, and Deliverable to end-users) criteria stated by the World Health Organization (WHO) for experimental procedures aimed at cancer screening and detection. Therefore, there is a continuing and pressing medical need to develop non-invasive screening tests for early detection that would be compatible with these requirements.

Unprecedented opportunities for early stage cancer detection have recently emerged from the characterization of the personalized protein corona (PC), i.e., the protein cloud that surrounds nanoparticles (NPs) upon exposure to patients’ bodily fluids [9,10]. Taking advantage of the alterations in the human proteome originated by the growth and spread of cancer, NP-enabled blood (NEB) tests allow for the distinguishing of cancer patients from healthy subjects by characterization of the personalized PC [11]. Current versions of the NEB tests are based on the “direct” characterization of the personalized PC by sodium dodecyl sulfate-polyacrylamide gel electrophoresis (SDS PAGE) and liquid chromatography tandem mass spectrometry (LC-MS/MS) [12]. However, the laborious step of protein isolation makes this technique subject to a high inter-operator variability and therefore to low reproducibility and reliability of experimental data. To go beyond these constraints, “indirect” characterization of the personalized PC, which avoids isolating plasma proteins from NPs, would be mandatory. In the present work, we present an NEB test founded on the physical principle of magneto-Archimedes levitation (MagLev). MagLev is a low-cost, easy to handle, and reproducible technique that uses a high-intensity magnetic field to separate diamagnetic objects [13] (including a variety of cell lines, organoids, bacteria, and yeast as well as tissues) according to their densities. However, the potential of MagLev for cancer detection has been marginally explored to date. In this work, the MagLev NEB test is based on the analysis of the levitation profiles of PC-coated graphene oxide (GO) nanosheets that migrate along a magnetic field gradient in a paramagnetic solution of dysprosium (III) nitrate hydrate. For the test validation, we employed human plasma samples from 45 individuals, 15 of them being healthy volunteers and 30 affected by four cancer types, i.e., breast cancer, prostate cancer, colorectal cancer, and pancreatic ductal adenocarcinoma (PDAC). These four cancer types were selected according to mortality data [14]. Over the last 15 years (2007–2021) prostate cancer, colorectal cancer, and PDAC have continuously been the second, third, and fourth cancer-related deaths in men, while breast cancer, colorectal cancer, and PDAC are the second, third, and fourth leading sites in the same ranking for women. According to the American Cancer Society, these two triplets of cancers have been accounting for more than 30% of cancer-related deaths for both sexes since 2007.

MagLev exhibited strong efficacy in distinguishing patients affected by PDAC with high specificity, sensitivity, and a global classification accuracy of 93%. This is a promising result considering that too late detection is the most serious concern of PDAC that causes an extremely low 5-year survival rate of 5%. This small percentage depends on the fact that most of the patients (80–85%) are diagnosed with locally advanced or metastatic conditions, which precludes them from undergoing surgical resection which is still the only chance to increase the survival rate considerably [15].

## 2. Materials and Methods

### 2.1. Preparation of Graphene Oxide Sheets

Graphene Oxide (GO) aqueous solution was purchased from Graphenea (San Sebastián, Spain). GO solution (0.25 mg/mL) was subjected to sonication (Vibra cell sonicator VC505, Sonics, and Materials, Newton, CT, USA) to obtain homogenous GO sheets as described elsewhere [16].

### 2.2. Patients’ Enrolment and Blood Sample Collection

This work was approved by the Ethical Committee of the University Campus Bio-Medico di Roma (Prot. 10/12 ComEt CBM and further amendments for PDAC and colorectal cancer: Prot. 10.3(12).18 for breast and prostate cancer). The study group comprised 15 non-oncological subjects (healthy control), 5 breast cancer patients, 5 prostate cancer patients, 5 colorectal cancer patients, and 15 PDAC patients. All the relevant information about enrolled patients is reported in Table S1 in the Supplementary Materials. The inclusion criteria for the study were: age > 18 years; adequate renal function (creatinine < 1.5 mg/dL, blood urea nitrogen < 1.5 times the upper limit); previous personal medical history negative for neoplasticity; renal or liver disease or blood disorders; no previous chemotherapy or radiotherapy; absence of uncontrolled infections; and written, informed consent. All blood samples were collected after the diagnosis of the tumor and before the patient underwent any type of treatment for the disease (e.g., surgical resection, chemotherapy, radiotherapy, etc.).

### 2.3. Preparation of Graphene Oxide–Human Plasma (GO–HP) Complexes

GO–HP samples were prepared as previously described [17]. Briefly, 50 µL of GO solutions (0.25 mg/mL) were incubated with 20 µL of human plasma (HP) from healthy donors and patients diagnosed with PDAC, breast cancer, colorectal cancer, and prostate cancer at 37 °C for 1 h. Distilled water was added to each sample to reach a total volume of 100 µL.

### 2.4. Size and Zeta-Potential Experiments

Size and zeta-potential experiments were carried out using a Zetasizer Nano ZS90 (Malvern, UK). Size measurements were performed using Malvern micro cuvettes (ZEN0040), while zeta-potential measurements were performed by using a Dip Cell Kit (ZEN1002). Experiments were made in triplicate and the results are reported as average ± standard deviation of three independent measurements.

### 2.5. Synchrotron Small Angle X-ray Scattering

Synchrotron small single X-ray scattering (SAXS) measurements were performed at the Austrian SAXS station of the synchrotron light source ELETTRA (Trieste, Italy). Further details of SAXS measurements can be found elsewhere [18]. Correction for background, primary beam intensity, and detector efficiency were included in the analysis of SAXS patterns.

### 2.6. MagLev Technology

The MagLev device involved two N42-grade neodymium (NdFeB) coaxial square permanent magnets (2.5 cm length, 2.5 cm width, and 5.0 cm height, purchased from Magnet4less) which face each other through N poles, with a separation distance of *d* = 2.8 cm. The strength of the magnetic field was ~0.5 T on the surface of the magnets. The sample container was a plastic cuvette of 4 mL and 2.5 cm in height. A paramagnetic aqueous solution of Dysprosium (III) nitrate hydrate (salt purchased from Sigma-Aldrich, Inc. Merk KGaA, Darmstadt, Germany) was used, concentrated at 80 mg/mL. Then, each GO–HP sample was injected at the bottom of the cuvette with a syringe kept upright until the complete dissolution of the sample. When the whole sample volume lifted towards the surface and distributed homogenously, the cuvette was inserted between the magnets. The Maglev system can levitate a diamagnetic object in a paramagnetic solution when the magnetic and gravitational forces cancel each other out:

(1) $${\vec{F}}_{mag}\;+\;{\vec{F}}_{g}\;=\;0$$

where *F_mag_* depends on the magnetic susceptibility of the paramagnetic medium (*m*), the magnetic susceptibility (*s*) and the volume (*V*) of the diamagnetic object, the magnetic field (*B*), and the magnetic permeability of free space (0) are as follows:

(2) $${\vec{F}}_{mag}\;=\;\frac{\chi_{s}-\chi_{m}}{\mu_{0}}V\left(\vec{B}\;\cdot \;\vec{\nabla }\right)\vec{B}$$

and *F_g_* is the buoyancy-corrected gravitational force, i.e.:

(3) $${\vec{F}}_{g}\;=\;\left(\rho_{s}\;-\;\rho_{m}\right)V\vec{g}$$

where the sample density and medium density are respectively represented by *s* and *m*, and the gravity acceleration by *g*. When the final equilibrium (Equation (1)) is reached, the diamagnetic sample reaches a steady height (*h*) that depends on the density of the object in the paramagnetic solution:

(4) $$h\;=\;\frac{d}{2}\;+\;\frac{\left(\rho_{s}\;-\;\rho_{m}\right)g\mu_{0}d^{2}}{\left(\chi_{s}\;-\;\chi_{m}\right)4B^{2}}$$

Equation (4) collects the information related to the expression of the magnetic force (Equation (2)), the gravitational force (Equation (3)), and the geometry of the magnetic setup. Finally, image series of MagLev patterns (at a controlled temperature of 25 °C) of both steady components and precipitating populations were acquired with a Nikon D5600 camera (time-lapse mode, 1 frame per 20 s) and processed by custom Matlab (Mathwork, Portola Valley, CA, USA) scripts. Briefly, for each frame, the vertical intensity profile was computed by averaging the recorded intensity over a region of interest containing the inner part of the cuvette. Then, after background subtraction, profiles were normalized to the maximum detected intensity over a reference window to avoid undesired effects due to exposure variations.

## 3. Results

**Figure 1:**
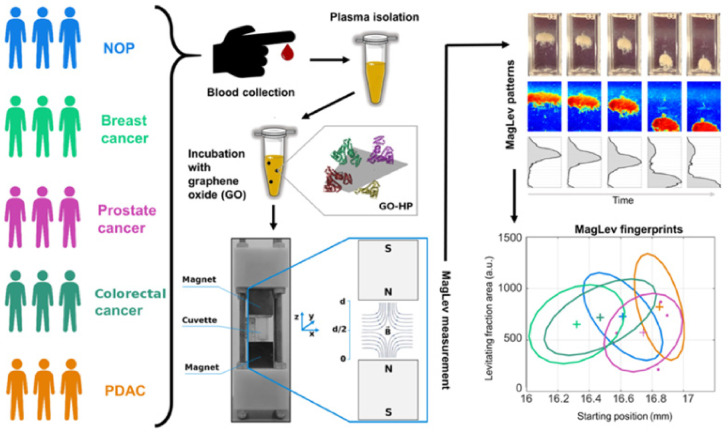
Scheme of the experimental workflow. Blood from donors belonging to 5 classes of physiological states was collected, then plasma was separated and incubated with graphene oxide (GO). GO–protein complexes were injected into a cuvette filled with a paramagnetic medium and inserted into a MagLev device. Levitation patterns were acquired, then processed to quantify MagLev profiles. Lastly, fingerprints for different cancer types were detected.

This study aimed to classify the MagLev fingerprints of personalized PC obtained by different classes of donors, namely non-oncological patients (NOP) and patients affected by breast cancer, prostate cancer, colorectal cancer, and pancreatic ductal adenocarcinoma (PDAC). The experimental workflow is depicted in Figure 1.

**Figure 2:**
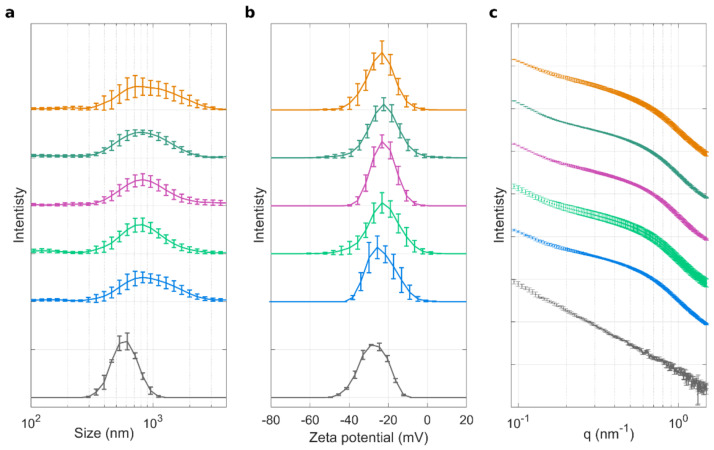
Characterization of GO sheets and GO–HP complexes. (**a**) Size distributions, (**b**) zeta potential distributions, and (**c**) synchrotron SAXS curves for bare GO (grey) and GO–HP samples obtained from NOP (blue), breast cancer (light green), prostate cancer (purple), colon cancer (dark green), and pancreatic ductal adenocarcinoma (PDAC) patients (orange). For each condition, each distribution is the average of five independent distributions, one for each of the single five donors.

Exposing GO to HP led to the formation of PC at the GO surface (i.e., GO–HP). Preliminary characterization of GO and GO–HP samples was performed in terms of size, zeta potential, and synchrotron SAXS curves. Results are reported in Figure 2a–c, respectively, for each of the investigated classes of donors. GO sheets were about 550 nm in size (Panel a, grey curve), and had negative zeta potential, i.e., −27 mV (Panel b, grey curve) and the mass fractal dimension by power-law synchrotron SAXS was 2.01 (Panel c, grey curve). According to the analysis of the SAXS pattern (Figure S1 in the Supplementary Materials), GO sheets were correctly described as 2-dimensional extended objects. Upon exposure to human plasma (HP), size and zeta potential increased, independently of the class of donors. Indeed, size distributions (Panel a, colored curves) were broader than that of bare GO (thus revealing a higher polydispersity) and peaked at about 850 nm. Zeta potential of GO–HP complexes was about −23 mV and SAXS patterns remarkably deviated from the bare GO reference, in the same way for all the classes of donors.

These measurements are compatible with protein binding to GO, leading to the formation of a protein corona and possible aggregation of GO sheets. Globally, results of Figure 2 indicate that it is not possible to classify non-oncological patients (NOP) and cancer samples simply by exploring the size, zeta potential, and SAXS profiles of GO–HP complexes.

**Figure 3:**
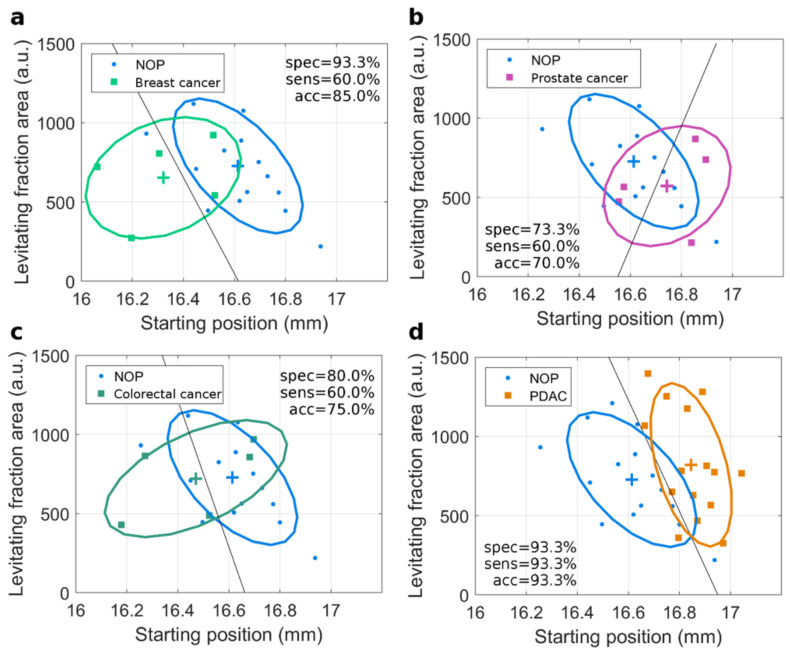
MagLev fingerprints for cancer. Scatterplots of detected MagLev fingerprints for (**a**) breast cancer, (**b**) prostate cancer, (**c**) colon cancer, and (**d**) PDAC. NOP samples are superimposed as references in all the panels. Mean values are reported as crosses, multivariate distributions are depicted by ellipses. Outputs from linear discriminant analysis (LDA) are indicated by solid lines. Test specificity, sensitivity, and global accuracy are listed in each panel. Specificity = number of correctly classified NOP/total number of NOP; sensitivity = number of correctly classified cancer patients/total number of cancer patients; accuracy = number of correctly classified subjects/total number of subjects.

Then, GO–HP samples were injected into a cuvette filled with a paramagnetic medium and inserted between the magnets of a MagLev device. Due to the combined effects of magnetic force, gravitational force, and buoyant force, GO–HP migrated in the cuvette originating a MagLev pattern, which was processed as described in Figure S2 in the Supplementary Materials. According to previous works [17], the starting position and the levitating fraction area at the longest acquisition time (i.e., 20 min) were identified as MagLev fingerprints and shown as scatterplots in Figure 3.

Each dot corresponds to a single donor and is the average value over three independent replicates. Class distributions are represented by ellipses, whereas crosses indicate the average values for each class (i.e., for each cancer type). Distributions of NOP are employed as a reference and are superimposed on each of the panels, corresponding to (a) breast cancer, (b) prostate cancer, (c) colorectal cancer, and (d) PDAC. The obtained disease-specific outcomes reflect the overlap of multivariate distributions with the NOP reference. The PDAC ellipse exhibited the minimum overlap with the NOP one, followed by the breast cancer distribution. Linear discriminant analysis (LDA) was performed to classify NOP and cancer patients, for each of the investigated diseases. Outputs are reported as black solid lines, which subdivide the parameter space into two regions. Samples were classified according to their location in one of those regions, and test specificity, sensitivity, and accuracy were evaluated for each specific disease (definitions are given in the caption of Figure 2). As Figure 3a clearly shows, NOP and PDAC distributions were well separated and yielded high values of specificity, sensitivity, and accuracy (i.e., 93%). This was mainly due to a significant change in the starting position. Conversely, breast cancer exhibited an opposite trend for the starting position, which was smaller for NOP (Figure 3b). In this case, although the LDA resulted in a good test specificity (93%), it misclassified 40% of the cancer patients, and thus the global accuracy was smaller (85%) than that obtained for PDAC classification (93%). Test specificity, sensitivity, and accuracy were also detected for the classification of prostate cancer (Figure 3c), and colon cancer (Figure 3d).

**Figure 4:**
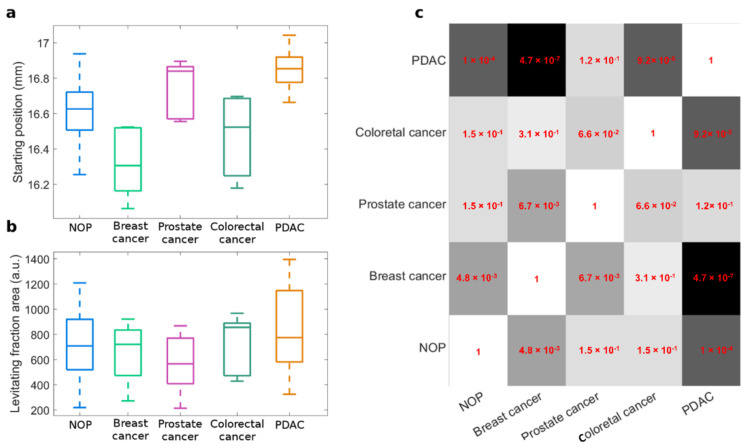
Statistical significance of MagLev fingerprints. Boxplots of MagLev fingerprints: (**a**) starting position, and (**b**) levitating fraction area. (**c**) Heatmap of *p*-values from Student’s *t*-test for the measured starting positions.

MagLev fingerprints were further analyzed by decoupling the single contributions. Distributions of sample starting positions and levitating fraction areas are reported separately in Figure 4a,b as boxplots. Inter-class variability was remarkably larger for starting positions (Figure 4a) than for levitating fraction areas (Figure 4b), whose 25th–75th-percentile-boxes were almost superimposable for all the diseases, except PDAC. As the measured starting positions were found to be the dominant contributions to the detected differences among classes, their statistical significance was evaluated by Student’s *t*-test and depicted as a heatmap in Figure 4c. By comparing the NOP distribution with all the other classes, the minimum *p*-values were found for PDAC and breast cancer, which read 10^−4^ and 4.8 · 10^−3^, respectively. Interestingly, the statistical significance of PDAC vs. breast cancer was even larger, with *p* = 4 · 10^−7^. Conversely, distributions of prostate cancer and colorectal cancer did not differ significantly from that of NOP, and neither do other cancer types. As the last step, a blind validation test was performed on a series of five NOP and five PDAC samples.

## 4. Discussion

To date, the early diagnosis of cancers is universally accepted as an essential factor for the success of health management, as it would enable healthcare providers to minimize the mortality of diseases, reduce the burden on society and improve the quality of life for patients and their families [1]. However, there is still a very limited number of non-invasive screening tests for early stage cancer detection for most cancer types. The efficacy of current medical protocols is still limited by diagnostic failures due to features of primary tumors (e.g., isodense tumors related to the surrounding parenchyma), to the patients (e.g., presence of metal implants), or adverse toxicity (e.g., nephrotoxicity, reactions to the contrast medium, radiation exposure) [17,19,20]. Nonetheless, PSA, the most useful marker for the diagnosis of prostate cancer, may not distinguish cancer from other prostate disorders, such as benign prostate hyperplasia and prostatitis [21]. Needing to be adjusted for the patient’s age, PSA may also underestimate severe pathological conditions [22]. Mammography represents the gold standard for breast cancer screening and early diagnosis. However, toxicity induced by the use of radiation and the risk of radiation-induced cancer in young women is not negligible [23]. Colonoscopy, strongly recommended for early colorectal cancer detection, is an invasive examination that can be burdened by severe complications, such as bowel perforation. Moreover, it can be limited in the detection of smaller (<10 mm) and flat polyps [24]. The urgent need for effective tools for cancer screening and detection pushed the scientific community to exploit the great potential of nanotechnology for diagnostic purposes. The study of the bio-nano interactions, i.e., the interactions between nanosized objects and biological systems, can provide unprecedented opportunities for the development of diagnostic technologies. When nanomaterials are exposed to biological fluids (e.g., HP, urine, saliva, gastrointestinal fluids, etc.), they become coated by a protein layer, referred to as the protein corona [25], whose composition is shaped by a complex interplay between the chemical-physical properties of the nanomaterial (e.g., size and surface charge [26]), the protein source (e.g., human plasma and its concentration [27]) and “environmental factors”, such as temperature [28], incubation time [29], and shear stress [30]. A series of papers by our group [10,31] demonstrated that the nanoparticle–protein corona is disease-specific, and personalized. This aspect paved the way for the development of sensor arrays for the discovery of cancer biomarkers [10], and nanoparticle-enabled blood tests. However, these approaches are based on analytical techniques (e.g., LC-MS/MS and 1D SDS-PAGE) that involve direct characterization of the PC by laborious steps for protein isolation and identification. For instance, the staining/destaining process used for protein visualization on 1D SDS PAGE after electrophoresis is user-dependent and leads to high variability in experimental outcomes. Globally, direct characterization of the protein corona is not aligned to the ASSURED criteria dictated by the WHO for cancer screening and detection and is hardly translatable into the clinic. To overcome these limitations, indirect characterization of the personalized PC (i.e., without isolating plasma proteins from NPs) is a crucial step. Recently, it has been demonstrated that MagLev is a faster, more manageable, and non-destructive platform that differentiates substances (e.g., drugs) for their densities. Since disease-specific PCs have different compositions and densities with respect to their “healthy counterpart”, their MagLev profiles along the magnetic field gradient may be distinguishable. In this proof-of-concept study, we asked whether the MagLev profile of corona-coated NPs could contain fingerprints of cancer. GO–HP samples were obtained by incubating GO nanosheets with HP from NOP, breast cancer, prostate cancer, colorectal cancer, and PDAC patients. These cancers were chosen according to the mortality data provided by the American Cancer Society. Indeed, these two triplets of cancer types (i.e., colorectal cancer, prostate cancer, and PDAC for men vs. colorectal cancer, breast cancer, and PDAC for women) have been continuously the second, third, and fourth leading causes of cancer-related deaths since 2007.

MagLev patterns of all the samples were acquired in triplicates and analyzed to detect potential disease-specific MagLev fingerprints. GO–HP samples in the MagLev platform exhibited a peculiar behavior. A large fraction precipitated at the bottom of the cuvette within 20 min, whereas a residual levitating fraction levitated in the paramagnetic medium. Based on recent literature [17], we focused on the starting position and the levitating fraction area. The analysis of MagLev profiles allowed for the distinguishing of cancer patients from NOP with a global accuracy in the order: PDAC > breast cancer > colorectal cancer > prostate cancer. Future studies will be performed to screen people at risk for PDAC, such as people with familiarity with this disease and/or subjects with obesity or diabetes, and to play a role in surveillance intervals.

As reported in Figure 4, the statistical significance of inter-class differences was significant only for PDAC vs. breast cancer. Alterations in the levitation patterns are due to different densities of NP–protein complexes that, in turn, are caused by changes in the structure and composition of the protein corona. Thus, the results reported in Figure 4 let us conclude that exposing GO to the plasma of NOP, PDAC, and breast cancer patients lead to personalized coronas of significantly different protein composition. According to the literature, we can speculate that these differences may be the consequence of alterations in the human proteome, resulting from mutations of genes that are known to be involved in the processes of carcinogenesis for both pancreatic and breast cancer (e.g., K-RAS and BRCA) [32]. Nonetheless, it has been recently reported that altered protein profiles in pancreatic and breast cancer occur and most of these proteins are involved in amino-acid, lipids, monosaccharides, and sulfur compound synthesis and metabolism [33].

Lastly, we explicitly comment on the limitations of the present investigation. The principal limitation of this investigation is represented by the relatively small sample size. We cannot exclude that the lack of statistical significance in the starting position of GO–HP between different cancer types (e.g., PDAC vs. prostate cancer; *p* = 0.12) could be due to the small sample size. On the other hand, computational studies [34] state that a large variance between two groups allows for the use of a regular *t*-test even with an extremely small sample size (*n* = 2) [35]. When we calculated the statistical significance between PDAC and breast cancer it returned a very low *p*-value of 4.7 · 10^−7^. Second, the poor representation in the sample of early stage PDAC patients seems to limit the relevance of our conclusions regarding the predictive ability of the test. However, the low rate of stage I and II PDAC is the real representation of the epidemiology of PDAC which is mostly detected at an advanced stage. Lastly, we cannot exclude that plasma proteins could undergo denaturation in dysprosium (III) nitrate hydrate. However, as clarified above, this is not a real weakness for diagnostic methods, whose main aim is the patients’ categorization and not the isolation of proteins in their active native state.

## 5. Conclusions

**Figure 5:**
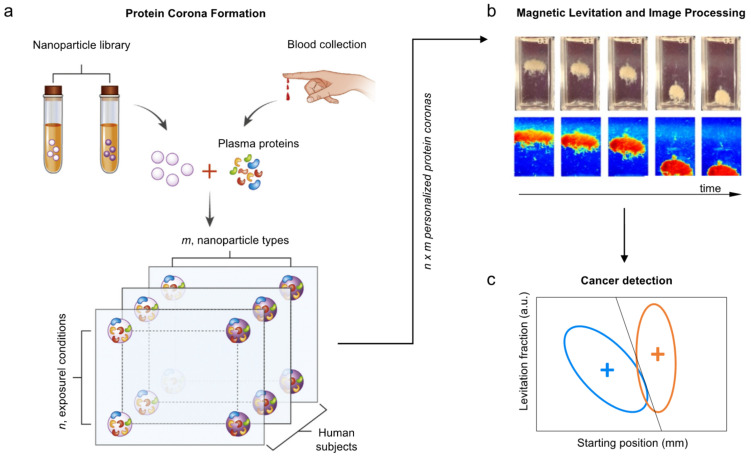
Optimization of the MagLev NEB test for cancer detection. (**a**) For a given cancer type, a library of *m* nanoparticle types is exposed to plasma proteins from patients with and without the condition under *n* different exposure conditions (e.g., protein concentration, incubation time, shear stress, etc.). (**b**) Each library component is introduced in the MagLev device and the levitation profile is followed in time. Image processing allows for the identification of a set of MagLev fingerprints for cancer (e.g., starting position of the nanoparticle–protein corona, the relative abundance of the levitating fraction, etc.). (**c**) Research is aimed at identifying one of the (*m* × *n*) library components that maximize sensitivity and the specificity of the MagLev NEB test. (Adapted from ref. [36]).

Using GO, human plasma, and 1 h incubation at 37 °C as model systems for the nanomaterial, the protein source, and the exposure condition, the developed MagLev NEB test was able to identify PDAC and breast cancer with high sensitivity and specificity. On the other hand, accuracy in identifying prostate cancer, and colorectal cancer was inadequate (<75%). As elsewhere specified [36], better performing versions of the MagLev NEB test may be easily achieved even for these cancer types (Figure 5). This methodology would benefit from the existence of almost endless combinations of tunable parameters, such as the intensity of magnetic field gradient, the paramagnetic medium, the chemical-physical properties of the nanomaterial, the protein source (e.g., human plasma, human serum, etc.), protein concentration and environmental factors (temperature, incubation time, shear stress, etc.). As MagLev is non-destructive testing, proteins isolated from the device could be analyzed by an analytical technique, such as LC MS/MS. While this does not fall within the scope of our investigation, this could represent a source of information to discover novel biomarkers of cancer growth and progression as well as to learn more about cancer biology. Beyond the conclusions of this proof-of-concept work, we envision our outcomes may pave the way for performing more in-depth investigations. When confirmed on a larger patient cohort, the MagLev NEB test could be clinically helpful at the first level of investigation to decide whether to perform more invasive exams and/or to follow up on oncological patients after surgery and/or pharmacological treatment.

## Data Availability

The data presented in this study are available on request from the corresponding author.

## Supplementary Materials

The following supporting information can be downloaded at: https://www.mdpi.com/article/10.3390/nano12091397/s1, Table S1: Demographic and clinic characteristics of the control group and cancers group (ASA: American Society of Anesthesiologists). Figure S1: SAXS curve for GO and corresponding fitting functions according to (a) Equation (1) and (b) Equation (2). Figure S2: Data acquisition and image processing. (a) Representative acquisition of image time series for MagLev analysis. (b) Schematic representation of image processing for a generic frame: a region of Interest (ROI) is set to contain the sample only. Vertical projection of the detected intensity is computed over the ROI, (c) frame by frame to obtain (d) the corresponding MagLev profiles. Profiles are normalized to a reference value that is evaluated as the average intensity on an area with uniform exposure. In this work, the investigated MagLev parameters for the determination of disease-specific fingerprints are the starting position of the samples and the levitating fraction area. The starting position is obtained as the intensity peak position at the first frame of the time series. The levitating fraction area is the integral area of the levitating peak at the last frame of the image time series (i.e., *t* = 20 min). References [37,38] are cited in the Supplementary Materials.
