# Supplementary material for: Magnetic Levitation of Personalized Nanoparticle–Protein Corona as an Effective Tool for Cancer Detection
Source: Nanomaterials (Basel). 2022 Apr 19;12(9):1397. doi: 10.3390/nano12091397 (PMC9104194; doi:10.3390/nano12091397)
Supplement: Supplementary file 1 [file nanomaterials-12-01397-s001.zip › nanomaterials-1673272-supplementary.pdf]

nanomaterials

MDPI

Supplementary Materials

Magnetic Levitation of Personalized Nanoparticle–Protein Corona as an Effective Tool for Cancer Detection

Erica Quagliarini [1,†], Luca Digiacomo [1,†], Damiano Caputo [2,3], Alessandro Coppola [3], Heinz Amenitsch [4], Giulio Caracciolo [1] and Daniela Pozzi [1,*]

[1] NanoDelivery Lab, Department of Molecular Medicine, Sapienza University of Rome, Viale Regina Elena 291, 00161 Rome, Italy; erica.quagliarini@uniroma1.it (E.Q.); luca.digiacomo@uniroma1.it (L.D.); giulio.caracciolo@uniroma1.it (G.C.)

[2] Department of Surgery, University Campus Bio-Medico di Roma, Via Alvaro del Portillo 200, 00128 Rome, Italy; d.caputo@policlinicocampus.it

[3] General Surgery, Fondazione Policlinico Universitario Campus Bio-Medico, Via Alvaro del Portillo 200, 00128 Rome, Italy; a.coppola@policlinicocampus.it

[4] Institute of Inorganic Chemistry, Graz University of Technology, Stremayrgasse 9/IV, 8010 Graz, Austria; amenitsch@tugraz.at

* Correspondence: daniela.pozzi@uniroma1.it

† These authors contributed equally to this work.

Table S1. Demographic and clinic characteristics of control group and cancers group (ASA: American Society of Anesthesiologists).

	**Controls (*n* = 20)**	**PDAC (*n* = 11)**	**Breast cancers (*n* = 5)**	**Prostate cancers (*n* = 5)**	**Colon cancers (*n* = 5)**
Median age, (range), y	66 (25–77)	69 (47–76)	71 (41–85)	66 (65–76)	64 (58–82)
Sex					
Male	11	6	0	5	4
Female	9	5	5	0	1
ASA score.					
I,	NA	–	2	0	0
II,	NA	3	2	5	5
III	NA	4	1	0	0
cT stage					
cT0	NA	0	0	0	0
cT1	NA	1	2	0	0
cT2	NA	2	3	5	0
cT3	NA	4	0	0	5
cT4	NA	4	0	0	0
cN stage					
cN0	NA	0	5	5	5
cN1	NA	5	0	0	0
cN2	NA	3	0	0	0
cN3	NA	3	0	0	0
cM stage				0	
cM0	NA	7	5	0	5
cM1	NA	4	0		0
pT stage					
pT0	NA	0	0	0	0
pT1	NA	1	1	0	0
pT2	NA	0	3	2	0
pT3	NA	3	1	3	5
pT4	NA	1	0	0	0
pTx	NA	2	0	0	0
pN stage					
pN0	NA	2	4	4	4
pN1	NA	1	0	1	1
pN2	NA	2	1	0	0
pN3	NA	–	0	0	0
pNx	NA	2	0	0	0
pM stage					
pM0	NA	5	5	5	5
pM1	NA	2	0	0	0
Stage					
I	NA	2	1	0	0
II	NA	2	2	3	4
III	NA	2	2	1	1
IV	NA	5	0	1	0
Missing	NA	0	0	0	0
Grading					
G1	NA	0	0	NA	0
G2	NA	6	0	NA	5
G3	NA	3	5	NA	0
Missing	NA	2	0	NA	0
Gleason Score					
G 6	NA	NA	NA	1	NA
G 7	NA	NA	NA	4	NA

G 8	NA	NA	NA	0	NA
G 9-10	NA	NA	NA	0	NA
CA 19.9, (UI/l)					
>37.00	0	8	NA	NA	0
<37.00	5	2	NA	NA	5
Missing	15	1	NA	NA	0
PSA (ng/ml)					
>4	NA	NA	NA	3	NA
<4	NA	NA	NA	1	NA
Missing	NA	NA	NA	1	NA

NA – not applicable.

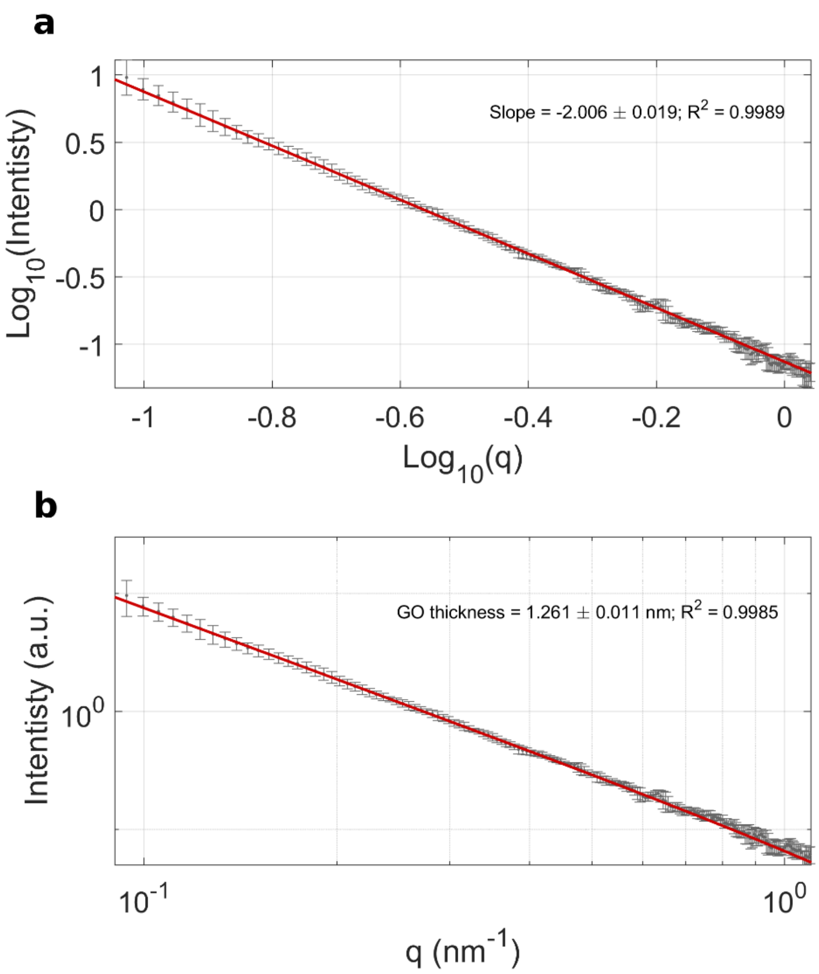

Figure S1. SAXS curve for GO and corresponding fitting functions according to (**a**) Equation (1) and (**b**) Equation (2).

Synchrotron SAXS data for GO was fitted by Equations (1) and (2).

$$\log_{10} I(q) = a_0 \log_{10} q + a_1 \quad (1)$$

$$I(q) = \frac{bT^2}{q^2}\left[\frac{\sin(qT/2)}{(qT/2)}\right]^2 \quad (2)$$

Equation (1) provides the mass-fractal dimension for GO [37], as the opposite of the slope, i.e., $-a_0 = 2$. This agrees with a mass-fractal power-law exponent for a thin, two-dimensional sheet. Equation (2) describes the SAXS curve of a flat object of thickness T

[38], which read about 1.3 nm. These arguments do not apply to GO-HP samples, which exhibited SAXS profiles very different from that of flat, two-dimensional systems.

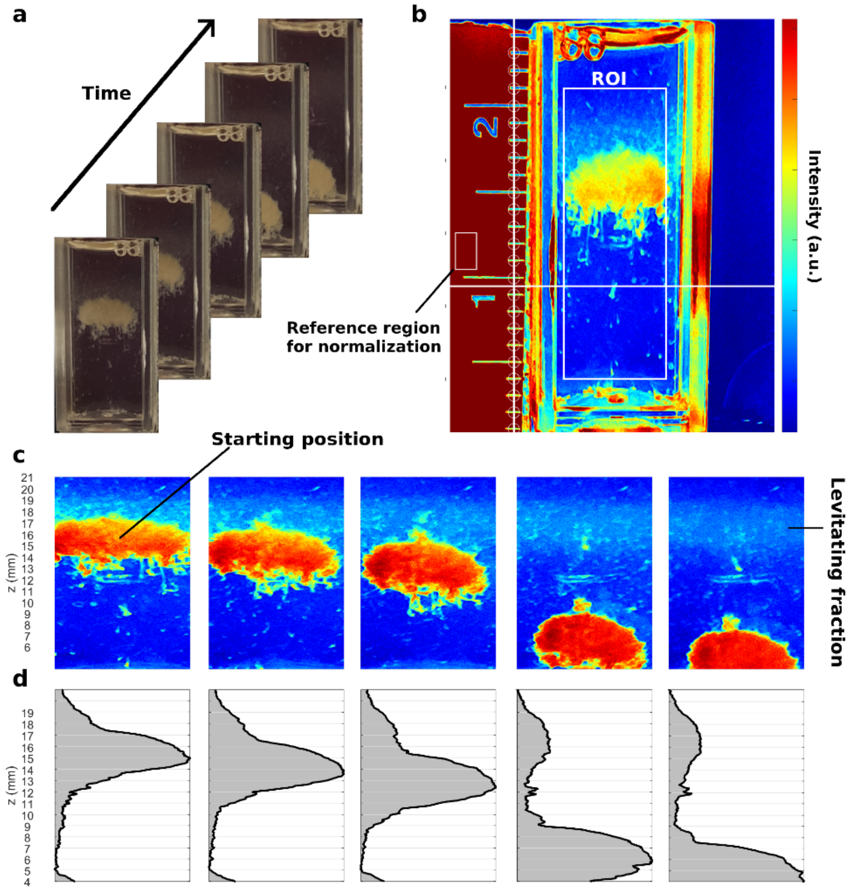

Figure S2. Data acquisition and image processing. (**a**) Representative acquisition of image time series for MagLev analysis. (**b**) Schematic representation of image processing for a generic frame: a region of Interest (ROI) is set to contain the sample only. Vertical projection of the detected intensity is computed over the ROI, (**c**) frame by frame to obtain (**d**) the corresponding MagLev profiles. Profiles are normalized to a reference value that is evaluated as the average intensity on an area with uniform exposure. In this work, the investigated MagLev parameters for the determination of disease-specific fingerprints are the starting position of the samples and the levitating fraction area. The starting position is obtained as the intensity peak position at the first frame of the time series. The levitating fraction area is the integral area of the levitating peak at the last frame of the image time series (i.e., $t = 20$ min).
